# Nano-/Micro-confined Water in Graphene Hydrogel as Superadsorbents for Water Purification

**DOI:** 10.1007/s40820-019-0336-3

**Published:** 2019-12-12

**Authors:** Yiran Sun, Fei Yu, Cong Li, Xiaohu Dai, Jie Ma

**Affiliations:** 1 State Key Laboratory of Pollution Control and Resource Reuse, College of Environmental Science and Engineering, Tongji University, 1239 Siping Road, Shanghai, 200092 People’s Republic of China; 2 College of Marine Ecology and Environment, Shanghai Ocean University, Shanghai, 201306 People’s Republic of China; 3 Department of Chemical and Biomolecular Engineering, The University of Akron, Akron, OH 44325 USA; 4 Research Center for Environmental Functional Materials, College of Environmental Science and Engineering, Tongji University, 1239 Siping Road, Shanghai, 200092 People’s Republic of China; 5 Shanghai Institute of Pollution Control and Ecological Security, Shanghai, 200092 People’s Republic of China

**Keywords:** Confined water, Graphene hydrogel, Hydrogen bonding, Adsorption, Antibiotic

## Abstract

Confined water has been proven to be of great importance due to its pervasiveness and contribution to life and many fields of scientific research. However, the control and characterization of confined water are a challenge. Herein, a confined space is constructed by flexibly changing the pH of a graphene oxide dispersion under the self-assembly process of a graphene hydrogel (GH), and the confined space is adjusted with variation from 10.04 to 3.52 nm. Confined water content in GH increases when the pore diameter of the confined space decreases; the corresponding adsorption capacity increases from 243.04 to 442.91 mg g^−1^. Moreover, attenuated total reflectance Fourier transform infrared spectroscopy and Raman spectroscopy are utilized to analyze the hydrogen bonding structure qualitatively and quantitatively, and correlation analysis reveals that the improvement in the adsorption capacity is caused by incomplete hydrogen bonding in the confined water. Further, confined water is assembled into four typical porous commercial adsorbents, and a remarkable enhancement of the adsorption capacity is achieved. This research demonstrates the application potential for the extraordinary properties of confined water and has implications for the development of highly effective confined water-modified adsorbents.

**Electronic supplementary material:**

The online version of this article (10.1007/s40820-019-0336-3) contains supplementary material, which is available to authorized users.

## Highlights

- Confined space/water in graphene hydrogel was constructed and controlled.
- Adsorption capacity of porous adsorbents was enhanced by filling confined water.
- Incomplete hydrogen bonding in confined water contributes to adsorption.

**Electronic supplementary material:**

The online version of this article (10.1007/s40820-019-0336-3) contains supplementary material, which is available to authorized users.

## Graphical abstract

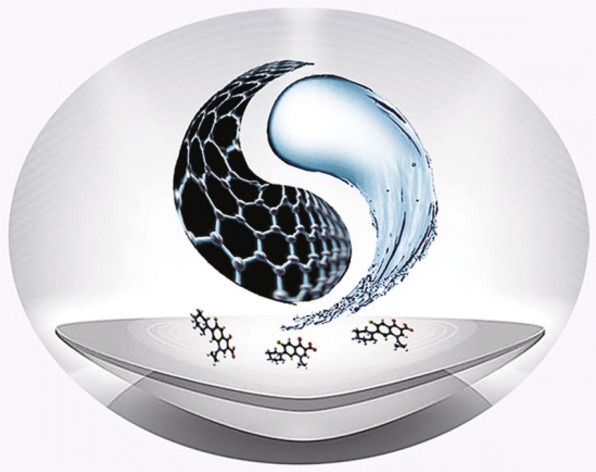

## Introduction

Water, as a major component of cells and a participant in life activities, is simple but crucial for all living things. Although water science research has been performed for thousands of years, our understanding of the science underlying its behavior and function needs to be strengthened [1]. Water exists in the form of bulk water and confined water [2]. Confined water generally refers to liquid water held within nanometer-sized vessels. It is found widespread in granular and porous materials, around and within cells, macromolecules, supramolecular structures, and gels [3]. Confined water is widespread in many kinds of systems in chemistry, biology, geology, materials science, and technology. Its unusual properties, including its hydrogen bonding structure and dynamic and thermodynamic behaviors, have extensively drawn the attention of scientists [4–6]. Confined water in and around proteins plays a crucial role in protein assembly and function [7]. Similarly, water confined in carbon nanomaterials, such as one-dimensional (1D) nanotubes and two-dimensional (2D) graphene, possesses outstanding properties [8–10]. Wu et al. trapped water in graphene sheets and found that the nano-confined water formed 2D square ice at room temperature. This is a new symmetric water phase in which the hydrogen bonding is qualitatively different from the conventional tetrahedral coordination between water molecules [11]. Additionally, the transport of water and guest molecules in confined water also differs from that in bulk water. Radha et al. fabricated 2D graphene capillaries and found that water transport through channels increased unexpectedly (up to 1 m s^−1^), which was attributed to high capillary pressures (approximately 1000 bar) and large slip lengths [12]. These extraordinary structures and properties endow it with the potential for applications in separation, nanochannel and nanofluidic devices, and desalination [13–15].

However, most previous researches have focused on the theoretical study of confined water, whereas studies on the application of confined water are lacking. In most cases, the preparation of confined water has been complex. The confined space is assembled first, and then, water enters the prepared confined space by vapor adsorption or high-pressure injection [8, 16]. The preparation of reverse micelles is simple, and it provides convenience for simulating confined water in biological systems. However, the mixtures of water, surfactants, and nonpolar solvents are not suitable for investigating the application of confined water [17]. In addition, due to its convenience under ideal conditions, molecular dynamics (MD) simulation has promoted the rapid development of theoretical studies for confined water. However, MD simulation has also limited the application of confined water, because simulated conditions are usually oversimplified compared with the conditions in practical situations. In addition, much focus has been given to the anomalous properties and functional mechanisms of confined water under extreme conditions, such as ultralow temperatures [18–20], and studies based on its application are still insufficient.

Due to their extraordinary 2D structure, graphene-based materials are usually chosen to conduct studies of confined water. Li et al. designed a confined 2D water path with a foldable graphene oxide (GO) film and used it to build an efficient solar desalination device with an efficiency reaching 80% under one-sun illumination [14]. Yang et al. developed a group of supercapacitors with multilayered graphene films, with water confined in graphene sheets acting as an effective spacer to prevent restacking [21]. Given the outstanding properties of confined water in graphene-based materials, GO has been selected to construct and adjust the skeleton of the confined space. Our group has found a water-enhanced adsorption phenomenon and revealed its mechanism based on buried water in a graphene hydrogel [22]. However, the definition and characterization of confined space and confined water are lacking. Therefore, an in-depth study of confined space, confined water, and adsorption performance needs to be conducted urgently.

So far, many commercial porous adsorbents with high specific surface area (SSA) have been developed [23, 24]. However, the adsorption capacity of contaminates in aqueous environment is restricted due to the underutilization of micropores. For example, the SSA of commercial activated carbon is approximately 2000 m^2^ g^−1^, but its adsorption capacity is relatively low due to poor surface accessibility for the micropores [25–28]. Chemical modification can effectively improve its adsorption capacity, while the chemical reaction in the modification process is complicated, costly, and pollution causing [29, 30]. Many studies have reported that the structure and amount of hydrogen bonding in confined water differ substantially from that in bulk water, which has led to unusual properties [31]. Additionally, hydrogen bonding is affected by the confined space structure and environment, such as pore size, surface wettability, and temperature [32, 33]. Therefore, the exploration of hydrogen bonding in confined water in a varied confined space may provide guidance to the application of confined water in improving the adsorption capacity of commercial adsorbents.

In this study, a confined space was constructed via the self-assembly of a graphene hydrogel (GH) and adjusted by changing the GO dispersion pH, which enabled adjustment of the confined water. The hydrogen bonding structure of water in the GH structure was qualitatively and quantitatively analyzed. Variation in the confined water significantly affected the adsorption performance of ciprofloxacin (CIP) onto GH. Correlation analysis revealed the relationships and influencing mechanism among the confined space, confined water, and adsorption capacity. Moreover, confined water was filled into several commercial porous adsorbents, and a remarkable enhancement of the adsorption capacity was achieved. It is believed that this work can provide guidance for developing highly efficient adsorbents and for facilitating the application of the extraordinary properties of confined water.

## Experiments

### Preparation of Graphene Hydrogel

Graphite oxide was synthesized according to the modified Hummers’ method [34]. The graphite oxide was dispersed in deionized water and sonicated for 6 h to obtain the GO aqueous dispersion (3.0 mg mL^−1^), which was then mixed with sodium ascorbate at a mass ratio of 1:2.2 and ultrasonicated to form a uniform dispersion. The pH of the dispersion was adjusted to 1.5, 3.5, 5.5, 8.5, and 12 by adding NaOH and HCl. A 4 mL dispersion was loaded in a glass vial and heated at 90 °C for 3 h without any disturbance. The GHs were named as GH-1.5, GH-3.5, GH-5.5, GH-8.5, and GH-12, respectively. Graphene aerogel (GA) was prepared by freezing the sample in − 20 °C for 5 h and drying it for another 48 h. All abbreviations appearing in this manuscript and their meanings are listed in Table S1.

### Batch Adsorption Experiments

The prepared GH samples were added to 20 mL CIP solutions with varying initial concentrations and shaken at a constant temperature of 25 °C at 150 rpm for a certain amount of time. Blank experiments without the GH samples were conducted to ensure that the decrease in concentration was caused by adsorption rather than volatilization or adsorption onto the bottle walls. All experiments consisted of at least two duplicate samples. After adsorption, the adsorbent samples were filtered and diluted for UV–visible spectroscopic measurements. Adsorption isotherm was studied in different concentrations (20, 40, 60, 70, 80, 90, 100, 120, 160, 200, and 300 mg L^−1^). Adsorption kinetics was studied in different reaction time (24, 48, 72, 96, 120, and 144 h) at initial concentration of CIP that is 150 mg L^−1^. The effect of pH was studied at different pH (2, 7, and 12), and the effect of ionic strength was studied at different NaCl concentrations (0, 0.1, and 1 mol L^−1^).

### Filling of Confined Water

The four porous materials, i.e., beta (SiO_2_/Al_2_O_3_, pore diameter: 0.55–0.7 nm), MOF (pore diameter: 0.8 nm), activated carbon (pore diameter: 2.0–2.2 nm), and CMK-13 (mesoporous carbon, pore diameter: 3.8–4.0 nm) were firstly dried and degassed in the vacuum drying chamber (80 °C, 2 h). Then, the samples were hydrated in a humid atmosphere at room temperature for 12 h. The moisture content is measured by the mass increment.

### Characterization Methods

The GA samples were characterized by scanning electron microscopy (SEM, Hitachi S-4800, Japan), transmission electron microscopy (TEM, JEOL2010F, 200 kV), X-ray diffraction (XRD, Bruker, D8 Advance), X-ray photoelectron spectroscopy (XPS, Kratos Axis Ultra DLD), Fourier transform infrared spectroscopy (FT-IR, NEXUS, 670), and SSA and aperture analysis (BELSORP-max-MicrotracBEL). The GH samples were characterized by attenuated total reflectance infrared spectroscopy (ATR-IR, Nicolet5700) and Raman spectroscopy (JOBIN-YVON LabRam 1B). All the Raman spectra correspond to an exciting laser wavelength of 532 nm. A UV–visible spectrophotometer (Techcomp UV2310 II) was used to determine the concentration of CIP based on the intensity of the peaks at 275 nm.

## Results and Discussion

### Characterization of Confined Space in GH

**Fig. 1:**
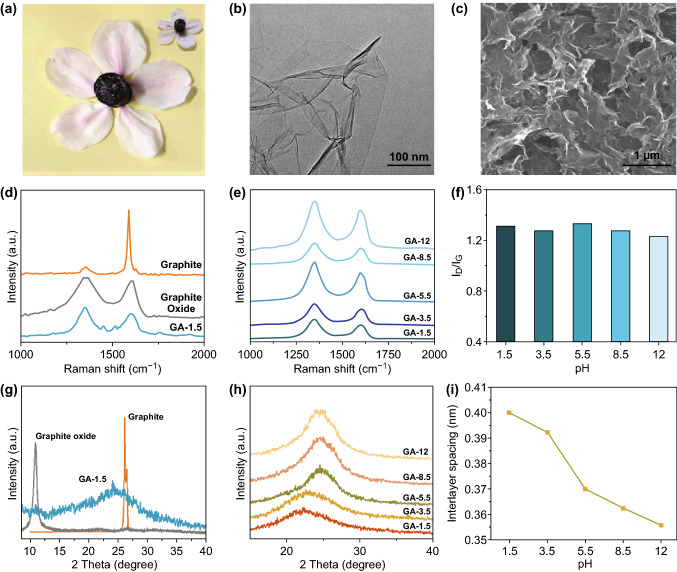
**a** Optical image of GH. **b** TEM, and **c** SEM images of GA; **d** Raman spectra of graphite oxide, graphene oxide (GO) and GA-1.5, **e** Raman spectra of the GA samples under different pH; **f** *I*_D_/*I*_G_ values for the GA samples; XRD patterns of **g** graphite, graphite oxide, GA-1.5, and **h** the GA samples; **i** the calculated interlayer spacing values for GA samples under different pH

GH samples were cylinder shaped, as shown in Fig. 1a. And 3 GHs (total aerogel mass: 0.0204 g) were tough enough to hold a 500 g weight (Fig. S1). TEM images exhibit several layers of graphene sheets in the GA structure (Fig. 1b), and many mesopores and micropores were formed due to the accumulation and interconnection of these graphene sheets, as shown in Fig. 1c. AFM images show that the thickness of graphene sheets in GA is ~ 4.4 nm, implying the few-layered graphene structure (Fig. S3). The *D* peak in the Raman spectra at approximately 1350 cm^−1^ represents the defective lattice structure of the carbon material, and the *G* peak at approximately 1620 cm^−1^ is caused by *sp*^2^ bonding. *I*_D_/*I*_G_ reflects the graphitization degree of the carbon material [35]. In Fig. 1d, the *I*_D_/*I*_G_ of GO (0.94) was much higher than that of graphite (0.17), revealing that the defects and the graphitization degree increased after oxidation. After chemical reduction, the *I*_D_/*I*_G_ of GA-12 further increased to 1.23, illustrating that graphene sheets randomly accumulated during the self-assembly process. Nonetheless, the *I*_D_/*I*_G_ ratios of five GA samples were similar, indicating that the manipulation of pH had a negligible effect on the number of defects and the graphitization degree of GH (Fig. 1e, f).

Figure 1g shows that graphite oxide and graphite had sharp peaks at 2*θ* = 11.0° and 26.5°, respectively, which implies that the interlayer spacing increased from 0.345 (graphite) to 0.807 nm (graphite oxide) due to the increased oxygen-containing functional groups on the graphite oxide sheets. However, the XRD pattern for GA-1.5 exhibited a broad and weak peak at approximately 2*θ* = 24.0°, which was attributed to the random accumulation of graphene sheets during the self-assembly process. The XRD patterns for all five GH samples showed a broad peak, and the shapes and intensities of the peaks were similar (Fig. 1h), while the location of the peaks shifts to the right with increasing GO dispersion pH. In Fig. 1i, the corresponding interlayer spacing for the GH samples decreased, illustrating that the GH structure became more compact.

**Fig. 2:**
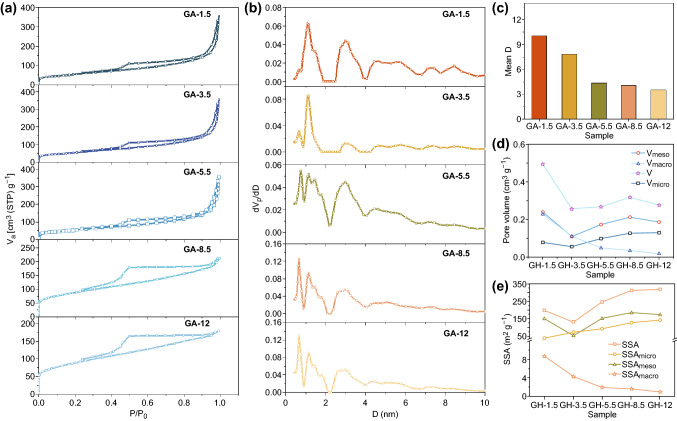
**a** N_2_ adsorption/desorption isotherms, **b** pore size distribution, **c** mean pore diameter, **d** pore volume, and **e** SSA of the GA samples

The N_2_ adsorption–desorption isotherms for the GA samples under different pH (Fig. 2a) exhibited Type IV curves, and hysteresis loops were obviously observed. As the GO dispersion pH increased, the hysteresis loop became bigger, and the volume adsorbed increased when *P*/*P*_0_ < 0.1, indicating an increased number of mesopores and micropores. Therefore, the SSA increased with the GO dispersion pH (Table S2). The pore size distribution (Fig. 2b) further displayed a variation in pores with the GO dispersion pH. The peaks at 2 < *D*<10 were assigned to mesopores. As the GO dispersion pH increased, the intensity of these peaks diminished, and some of them faded. The intensity of the broad peak at 1 < *D*<2, which is assigned to the micropores, also decreased. In comparison, the peak at approximately *D *= 0.7 nm in GA-1.5 was fairly weak, but the intensity increased significantly with increasing GO dispersion pH. Overall, when the GO dispersion pH was increased, more micropores were found to be present, while the number of mesopores decreased. The calculated mean pore diameter of the GA samples (Fig. 2c) also reduced in accordance with the variation in the interlayer spacing (Fig. 1i).

To further investigate the pore structure variation, we calculated the pore volume and SSA of micro-, meso-, and macropores (Fig. 2d, e). With increasing GO dispersion pH, *V*_macro_ (i.e., the pore volume of the macropores) and SSA_macro_ (i.e., the SSA of the macropores) were reduced, while *V*_micro_ (i.e., the pore volume of the micropores) and SSA_micro_ (i.e., the SSA of the micropores) showed a significant increase. This observation verified the decrease in macropores and increase in micropores, which agreed with the pore size distribution. In addition, both *V*_meso_ (i.e., the pore volume of the mesopores) and SSA_meso_ (i.e., the SSA of the mesopores) first decreased in GH-3.5 and then increased, which was ascribed to the fact that the 2–4 nm pores in GH-3.5 were much smaller than those in the other samples (Fig. 2b). For *V* (i.e., the pore volume of GH) and SSA (i.e., the SSA of GH), they showed almost the same trend as that of the mesopores, indicating the predominate nature of mesopores in GH samples. Moreover, the finding that macropores contributed more to the pore volume while micropores affected the SSA more was also evident.

**Fig. 3:**
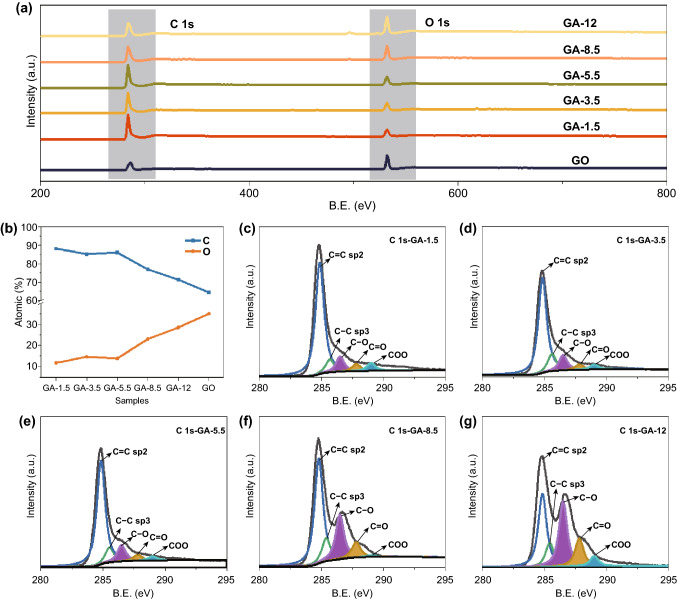
**a** XPS spectra of GA samples and GO, **b** percentages (at%) of C and O in the GA samples and GO, and C 1s core level spectra for GA samples: **c** GA-1.5, **d** GA-3.5, **e** GA-5.5, **f** GA-8.5, and **g** GA-12

XPS and FT-IR were utilized to investigate the variation in the surface characteristics of confined space in GH (Figs. 3 and S2). The presence of C and O in XPS survey spectrum (Fig. 3a) revealed the simple composition of the confined space. In Fig. 3b, the percent of O in GO (35.13 at%) was much higher than that in GA samples (11.74 to 28.55 at%). Additionally, the intensity of the hydroxyl groups (3400 cm^−1^), carboxyl and carbonyl groups (1730 and 1610 cm^−1^) in GA samples was significantly weaker than that in GO (Fig. S2a) [36]. The results indicated the reduction of oxygen-functional groups in the self-assembly process. Moreover, with increasing dispersion pH of GO, the percent (at%) of O increased from 11.74 to 28.55 at% (Fig. 3b), which was in agreement with the color change observed in the residual solution after reaction (Fig. S4), illustrating that higher pH restrained the degree of chemical reduction in the self-assembly process.

The peaks for the GA samples prepared at different GO dispersion pH values did not show an obvious difference (Fig. S2b); thus, the kind of oxygen-containing functional groups in these samples were similar. The C 1s spectra further demonstrated the variation in the oxygen-functional groups, as shown in Fig. 3c–g. It can be clearly observed that with increasing GO dispersion pH, there was a significant increase in the areas of the peaks corresponding to the carbon–oxygen single bond (286.5 eV), carbon–oxygen double bonds (287.8 eV), and carboxyl groups (289.0 eV), indicating that the relative contents of these oxygen-containing functional groups in GA were increased. This phenomenon was regarded as the main reason for the decrease in reduction degree [37].

Given the above analysis, the confined space in GH was adjusted successfully by simply changing the GO dispersion pH. The adjustment changed the pore size and the surface oxygen-functional groups of GA violently. When the GO dispersion pH was increased, the graphene sheets combined more tightly (Fig. 1h), and the interlayer spacing (Fig. 1i) and the pore size (Fig. 2c) decreased. More micropores emerged, and the SSA increased. Additionally, with increasing the GO dispersion pH, the reduction degree decreased, and the oxygen-functional groups on the GA surface increased (Fig. 3). The adjustment of the confined space in GH had a significant effect on the confined water, which is discussed in the next part.

### Qualitative and Quantitative Analysis of Confined Water in GH

**Fig. 4:**
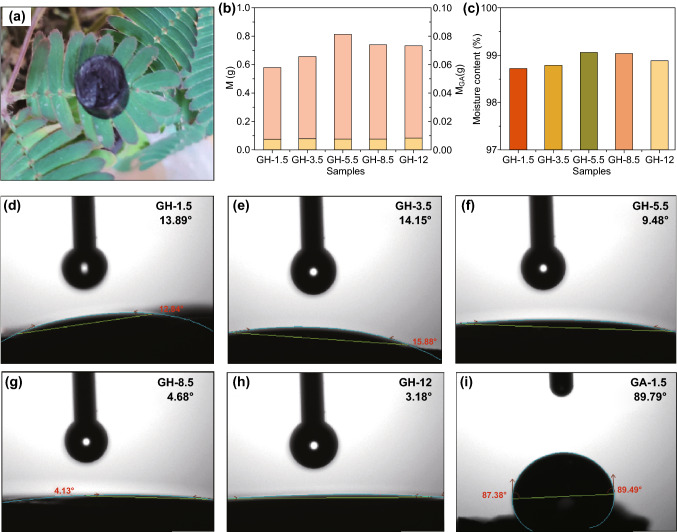
**a** Optical image of GA, **b** a quality comparison of the GA and GH samples, and **c** the moisture content of the GH samples. Contact angle of (**d**–**h**) the GH samples and **i** GA-1.5

In Fig. 4a, the GA was placed on the leaf of *Mimosa pudica*; the leaf did not close up, indicating that the GA had a very low density (0.0068 g cm^−3^). The GA is the skeleton of the GH and was used to construct the confined space for holding water in the GH structure. The masses of all the GHs were much higher than that of the GA, and the moisture content of all the GHs was all greater than 98.5% (Fig. 4b, c). Interestingly, the *M*_GH_ (i.e., the quality of one GH sample), *M*_GA_ (i.e., the quality of one GA sample) and moisture content showed the same trends: increasing first and then decreasing with increasing GO dispersion pH. Their maximum values were reached when pH = 5.5.

The contact angles were evaluated to investigate the surface wettability of the GH and GA samples (Figs. 4d–i and S5). All GH and GA samples were hydrophilic because the contact angles were less than 90°, but the hydrophilicity of GH was much higher than that of GA. The surface wettability of GA samples was weak, for the contact angle of GA ranged from 89.79° to 71.06°. However, the contact angle of GH was between 14.15° and 3.18°, showing that GH was similar to a superhydrophilic surface. This phenomenon revealed that the presence of confined water in the GH structure could significantly improve its surface wettability, which benefits access to contaminants in the GH structure. Interestingly, contact angles of both GA and GH decreased with increasing the GO dispersion pH. This property was consistent with the variation in the oxygen content observed via XPS (Fig. 3b), as more oxygen-containing groups were retained on the graphene sheet surface, and thus, the hydrophilicity of GA was increased.

The difference in hydrogen bonding between confined water and bulk water is a remarkable feature, which can be characterized by the O–H stretching band in the IR [16, 38–40]. Moreover, different categories of hydrogen bonding structures can be classified and analyzed based on the Raman spectrum [41–44]. In addition, both ATR-IR and Raman spectra can be used to realize in situ measurements and provide direct information for the confined water in a GH. Therefore, ATR-IR and Raman spectra were chosen to verify and quantitatively analyze the existence of confined water in the GH structure.

**Fig. 5:**
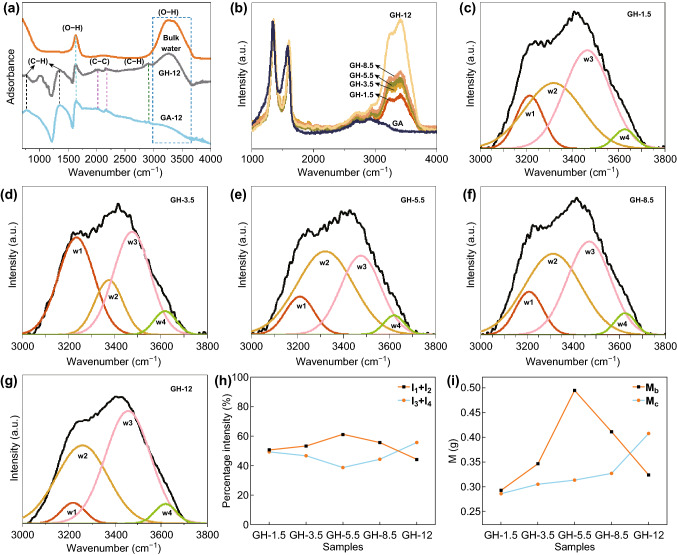
**a** ATR-IR spectra for bulk water, GH-12, and GA-12; **b** Raman spectra of the GA and GH samples; **c**–**g** OH stretching sub-band of water in the GH samples; **h** percentage intensity of *w*_1_ + *w*_2_(*I*_1_ + *I*_2_) and *w*_3_ + *w*_4_(*I*_3_ + *I*_4_) in the GH samples; and **i** mass of bulk water (*M*_b_) and confined water (*M*_c_) in the GH samples

ATR-IR results for GH-12, GA-12, and bulk water are shown in Fig. 5a. For GA-12 and GH-12, the spectra both showed peaks caused by the bending vibration (612 cm^−1^) and stretching vibration (1420 cm^−1^) of the carbon–hydrogen bond and the skeletal vibration of the benzene ring (2000 and 2200 cm^−1^), respectively, indicating that the structures of GH and GA were similar. However, the intensity of the carbon–oxygen double bonds of the carbonyl groups (1620 cm^−1^) in GH-12 was stronger than that in GA-12, which was assigned to the presence of the bending vibrations of hydrogen bonds in water [45, 46]. Moreover, comparing the peaks of the stretching vibration of the hydroxy groups from 3000 to 3700 cm^−1^ in GH-12 with those of bulk water, the location of these peaks in GH-12 shifted toward lower wavenumbers, and the shape of the broad peak changed. This provided significant evidence for the existence of confined water [16, 40], indicating that the hydrogen bonding structure of GH was different from that of bulk water. In addition, the broad and strong peak from 3000 to 3700 cm^−1^ in the Raman spectra was found in the GH samples but not in the GA (Fig. 5b) samples, which also verified the presence of confined water [47].

To investigate how confined water in the GH samples varied with confined space, we carried out further Raman spectrum measurements and normalized them by the *D* peak of the graphene sheets (Fig. 5b). It was significant that the intensity of the hydrogen bonding in GH (3000–3700 cm^−1^) increased with the GO dispersion pH [47] and that the intensity of the subpeak at the higher wavenumber became stronger. Additional quantitative analysis was conducted by carrying out a deconvolution procedure for the OH stretching band.

As reported in the literatures [42, 43], the OH stretching band can be decomposed into four different spectral components corresponding to four classes of hydrogen bonding structures. The sub-band at the lowest wavenumber (approximately 3220 cm^−1^, *w*_1_) was assigned to the symmetric OH stretching modes of the tetrahedral hydrogen bonding networks, and the sub-band at approximately 3350 cm^−1^ (namely *w*_2_) represented the asymmetric OH stretching modes of the tetrahedral hydrogen bonding networks. The sub-band at approximately 3450 cm^−1^ (namely *w*_3_) reflected the not-in-phase OH stretching mode of the distorted tetrahedral hydrogen bonding networks, and the highest wavenumber sub-band at approximately 3610 cm^−1^ (namely *w*_4_) was consistent with the OH stretching mode of the broken hydrogen bonding networks. The percentage contents of the four categories of hydrogen bonding structures were *I*_1_, *I*_2_, *I*_3_, and *I*_4_. According to the definition of the four categories of hydrogen bonding and the description reported in published papers [44], the percentage content of *w*_1_ + *w*_2_ (*I*_1_ + *I*_2_) was assigned to the hydrogen bonding in bulk water due to the hydrogen bonding being tetra-coordinated, while the percentage content of *w*_3_ + *w*_4_ (*I*_3_ + *I*_4_) represented the hydrogen bonding in confined water because this kind of hydrogen bonding was perturbed by the interface of the confined space.

The fitted results for the OH stretching mode of the GHs are shown in Fig. 5c–g. Figure 5h displays the evolution of the content of hydrogen bonding in bulk water (namely *I*_1_ + *I*_2_) and hydrogen bonding in confined water (namely *I*_3_ + *I*_4_) as a function of the GO dispersion pH, representing the variation between different hydrogen bonding structures. It appeared that the percentage intensity of *w*_1_ + *w*_2_ (i.e., the content of hydrogen bonding in bulk water) firstly increased before reaching a maximum for pH = 5.5 and then decreased with increasing GO dispersion pH. Correspondingly, the content of *w*_3_ + *w*_4_, which represents the hydrogen bonding in confined water, displayed adverse trend with a minimum at pH = 5.5. Combined with the analysis in Fig. 4b, c, we found that the moisture content, *M*_GH_, *M*_GA_, showed the same trend with *I*_1_ + *I*_2_, illustrating that the hydrogen bonding in bulk water increased with the total mass of the GH. The mass of the bulk (*M*_b_, (*M*_GH_ − *M*_GA_) × (*I*_1_ + *I*_2_)) and confined water (*M*_c_, (*M*_GH_ − *M*_GA_) × (*I*_3_ + *I*_4_)) in one GH sample (Fig. 5i) were also calculated, and it was observed that *M*_c_ continued to increase with increasing GO dispersion pH. Therefore, the confined water in the GH samples was successfully controlled by adjusting the GO dispersion pH.

### Adsorption Capacity of GH for CIP for Different Levels of Confinement

**Fig. 6:**
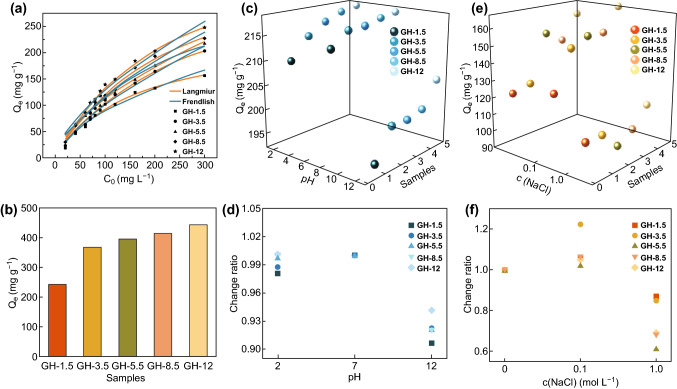
**a** Equilibrium adsorption isotherms for the GH samples, **b** maximum adsorption capacity of the GH samples calculated by Langmuir isotherms, **c** adsorption capacity, and **d** change ratio of the adsorption capacity for the GH samples under different *c*(NaCl) conditions. **e** Adsorption capacity and **f** change ratio of the adsorption capacity of the GH samples prepared at different pH levels

According to the finding that *M*_c_ increased with increasing GO dispersion pH, we further conducted batch adsorption experiments to investigate the variation in the adsorption capacity for the confined water in GH. As shown in Fig. 6a, the adsorption capacity of GH samples for CIP increased with increasing GO dispersion pH. The correlation coefficients (*R*^2^) for the GH samples fitted via the Langmuir model were all higher than those fitted via the Freundlich model, revealing that the former model was more suitable for the adsorption process of CIP in GH (Fig. 6a, Table S3). Additionally, the maximum adsorption capacity of the GH samples (Fig. 6b) also increased from 243.04 to 442.91 mg g^−1^ with increasing GO dispersion pH. Therefore, the adsorption capacity of the GH samples was raised as the confined water content increased.

Then, the adsorption efficiency of GH samples under varying contact time, pH, and ionic strength was evaluated to assess the comprehensive adsorption capacity of GH samples with varying confined water content. As shown in Fig. S6, the GH samples prepared at different GO dispersion pH values did not show significant differences in adsorption rate, indicating that the confined water did not make a significant difference to the CIP transfer in GH samples. Both acidity and alkalinity decreased the adsorption capacity of all GH samples (Fig. 6c). After the adsorption data were normalized according to the adsorption capacity at pH = 7 (Fig. 6d), the adsorption capacity of GH-1.5 decreased by approximately 10% at pH = 12, while that of GH-12 decreased by only 5%. The decrease in the adsorption capacity for the other three GH samples was almost the same (approximately 7%). In addition, the adsorption capacity decreased by less than 2% at pH = 2, indicating that acidic conditions had a negligible effect on the adsorption capacity of the GH. These results indicated that the GH samples with more confined content showed better resistance to alkalinity and acidity. In Fig. 6e, the adsorption capacity of all GH samples increased slightly under low ionic strength conditions and decreased under high ionic strength conditions. In Fig. 6f, the adsorption capacity of GH-1.5 reduced by approximately 10%, while those of GH-8.5 and GH-12 decreased by approximately 30%, illustrating that GH samples with less confined water content showed better ionic strength resistance.

It has been reported that confined water is affected by the structure and surface properties of the confined space [32, 33]. Additionally, variation in the confined water can affect the properties in application [13–15]. According to the above results, confined water was adjusted by varying the confined space, and the CIP adsorption capacity of the GH was changed, as well. Therefore, we proposed two hypothetical explanations based on correlation analysis.

**Fig. 7:**
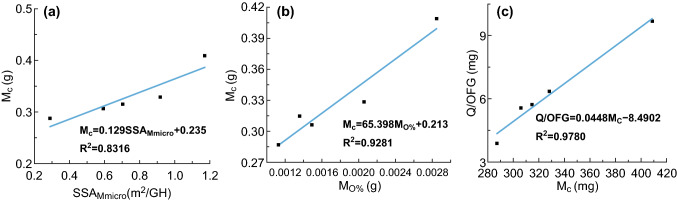
**a** The linear fitting results for SSA_Mmicro_ (SSA of the micropores in one GH sample) and *M*_c_ (mass of confined water in one GH sample); **b** the linear fitting results for *M*_o%_ (oxygen content in one GH sample) and *M*_c_; and **c** the linear fitting results for the *M*_c_ and *Q*/OFG (normalized adsorption capacity of the confined water for each GH sample)

We firstly conducted a simple fitting process to investigate the relationship between two important parameters of the GA (i.e., the SSA and oxygen content) and confined water. First, the three parameters were normalized by the mass of one GH sample, as shown in Table S4. The fitting results are presented in Figs. S7a–c and 7a. As presented in Fig. S7a, *M*_c_ (mass of confined water of one GH sample) exhibited a positive correlation with the SSA for each GH sample, but the correlation coefficient was low. Then, we attempted to fit *M*_c_ with the *SSA*s of the micropores, mesopores, and macropores. SSA_Mmicro_ (SSA of the micropores in one GH sample) showed the strongest linear positive correlation with *M*_c_, illustrating that the interface of the micropores dominated the variation in the confined water. We speculated that this result was due to the increase in the number of micropores, which increased the confinement degree. More hydrogen bonding existed in the form of distorted or broken structures, and thus, the content of confined water increased with increasing SSA_Mmicro_. Next, we fitted *M*_c_ with *M*_o%_ (oxygen content of one GH sample), as shown in Fig. 7b. *M*_c_ exhibited a strong linear relationship with the oxygen content. This phenomenon proved that the wettability of the interface between the water and graphene sheets affected the confined water content. With the increase in oxygen content, the hydrophilicity of the interface improved, and more water molecules tended to combine with the graphene sheets, which caused the content of confined water to increase.

In the second part, we conducted a correlation analysis for the CIP adsorption capacity of the GH samples with confined water and bulk water. To precisely measure the adsorption capacity of the three parts (i.e., confined space (graphene sheets), confined water, and bulk water) in GH, we first normalized the adsorption capacity of the graphene sheets followed by the consideration that the adsorption capacity of the GH for CIP consists of physical adsorption and chemical adsorption on graphene sheets and the adsorption of bulk and confined waters. CIP is adsorbed on graphene sheets via hydrogen bonding and *π*–*π* electron donor–acceptor (EDA) interaction. In hydrogen bonding between graphene sheets and CIP, the –C=O, –NH_2_, and –OH in CIP act as electron donors, and –COOH in GH took on the role of electron acceptors, while in the *π*–*π* EDA interaction, the –OH in GH acts as an electron donor, while the nitrogen-containing heterocyclic ring and fluorine-containing benzene ring in CIP take on the role of electron acceptors [48, 49]. Therefore, the adsorption capacity was normalized according to the content of carboxyl and hydroxy groups in the GA (i.e., *Q*/OFG). Regression analysis of *Q*/OFG with *M*_c_ and *M*_b_ (mass of bulk water of one GH sample) was conducted, and the results are listed in Tables S5–S7. The value of *R*^2^ was 0.9924, indicating that their relationship had a high positive correlation. The value of significance *F* (0.015) was lower than 0.050, illustrating that the regression was significant. By comparing the *P* values of *M*_b_ (0.444 > 0.050) and *M*_c_ (0.0076 < 0.050), we concluded that *M*_c_ had a high positive correlation with *Q*/OFG; however, there was no significant correlation between bulk water and *Q*/OFG. Based on this result, we further conducted a linear regression analysis between *Q*/OFG and *M*_c_. The results (Fig. 7c) revealed that there was a significant positive correlation. By combining the regression equation with the three kinds of buried water in the adsorption mechanism, we speculated that the bulk water mainly provided the supporting media and transport channels in the GH and that these two roles did not vary with the graphene structure under the condition that there is no invalid stacking among the graphene sheets. Importantly, the hydrogen bonding role was mainly shown in confined water. Most of the hydrogen bonding was in the form of a distorted tetrahedral structure or a broken hydrogen bonding network, thus compensating for the increased chemical potential via the loss of some bonds and reduced competition from neighboring water molecules, which is caused by the restriction of the confined space. The strength of hydrogen bonding in the confined water increased the rate of the reaction with CIP molecules. Therefore, hydrogen bonding in confined water enhanced the adsorption capacity of the GH samples for CIP.

The strategy that manipulating the confined water is applied in other porous materials in this research, further enhancing the significance of confined water. The above experimental results indicated that the adsorption capacity of the GH for CIP increased with increasing confined water content. Therefore, we tried to apply confined water for the modification of conventional adsorbents. We chose four materials with different pore diameters and filled the pores with confined water. The adsorption capacity of the four materials with and without confined water is presented in Fig. S8. When filled with confined water, the adsorption capacity of these four adsorbents improved with different extent compared with dry samples. The filling of confined water is much cheaper and more eco-friendly than other chemical modification methods.

## Conclusion

This work conveniently constructed controllable confined space and confined water via changing the GO dispersion pH. The pore size and oxygen content of the confined space were adjusted successfully. The qualitative and quantitative analyses of the confined water revealed that the confined water content and hydrogen bonding structure were regulated with the variation in confined space. The fitting results showed that the SSAs of micropores and oxygen contents both contributed to the confined water content. Furthermore, it is illuminated that the increase in confined water promoted the adsorption capacity of CIP onto graphene hydrogels, and the enhancement was dominated by the increased strength of hydrogen bonding in confined water. Bulk water in GH constricted the aggregation of graphene sheets and provided channels for CIP transportation. Moreover, four common porous adsorbents were modified with confined water, and the adsorption capacity was improved. This work analyzed the enhancement mechanism of confined water on adsorption and provides a reference for improving the adsorption capacity of porous adsorbents.

## Electronic supplementary material

Below is the link to the electronic supplementary material.

Supplementary material 1 (PDF 410 kb)
