# Supplementary material for: Nano-/Micro-confined Water in Graphene Hydrogel as Superadsorbents for Water Purification
Source: Nanomicro Lett. 2019 Dec 12;12:2. doi: 10.1007/s40820-019-0336-3 (PMC7770964; doi:10.1007/s40820-019-0336-3)
Supplement: Supplementary file 1 — Supplementary material 1 (PDF 410 kb) [file 40820_2019_336_MOESM1_ESM.pdf]

Supporting Information for

Nano/Micro-Confined Water in Graphene Hydrogel as Superadsorbents for Water Purification

Yiran Sun[1], Fei Yu[2,] *, Cong Li[3], Xiaohu Dai[1, 5], Jie Ma[1, 4, 5,] *

[1]State Key Laboratory of Pollution Control and Resource Reuse, College of Environmental Science and Engineering, Tongji University, 1239 Siping Road, Shanghai 200092, People's Republic of China

[2]College of Marine Ecology and Environment, Shanghai Ocean University, Shanghai 201306, People's Republic of China

[3]Department of Chemical and Biomolecular Engineering, The University of Akron, Akron, Ohio 44325, USA

[4]Research Center for Environmental Functional Materials, College of Environmental Science and Engineering, Tongji University, 1239 Siping Road, Shanghai 200092, People's Republic of China

[5]Shanghai Institute of Pollution Control and Ecological Security, Shanghai, 200092, People's Republic of China

S1 Materials and Chemicals

All chemicals were purchased from Sinopharm Chemical Reagent Co., Ltd. (Shanghai, China) in analytical purity and used without further purification. All solutions were prepared using deionized water.

The adsorption capacity of CIP (Qe, mg g^{-1}) was calculated as Eq. S1:

$$\boldsymbol{Q_e = \frac{(C_0 - C_e)V}{m}} \qquad \text{(S1)}$$

Where C_0 represents the initial CIP concentration (mg L^{-1}), Ce represents the equilibrium CIP concentration (mg L^{-1}), V represents the solution volume (L), and m represents the weight of the adsorbent (g).

S2 Langmuir Model

The form of the Langmuir isotherm can be represented by Eq. S2:

$$q_e = q_m \frac{K_L C_e}{1 + K_L C_e} \qquad \text{(S2)}$$

where q_e is the adsorption capacity of contaminate (mg g^{-1}), C_e denotes the equilibrium concentration of contaminate in solution (mg L^{-1}); K_L represents the Langmuir constant (L mg^{-1}) that relates to the affinity of binding sites, and q_m is a theoretical limit of adsorption capacity when the monolayer surface is fully covered with contaminate molecules to assist in the comparison of adsorption performance (mg g^{-1}). Furthermore, the effect of the isotherm shape was studied to understand whether an adsorption system is favorable or not. Another important parameter, R_L, called the separation factor or equilibrium parameter, which can be used to determine the feasibility of adsorption in a given concentration range over adsorbent, was also evaluated from the relation:

$$R_L = \frac{1}{1 + K_L C_0} \qquad \text{(S3)}$$

where K_L is the Langmuir adsorption constant (l/mg) and C_0 is the initial contaminate concentration. Ho and McKay established that (1) $0< R_L <1$ for favorable adsorption; (2) $R_L>1$ for unfavorable adsorption; (3) $R_L=1$ for linear adsorption; and (4) $R_L=0$ for irreversible adsorption.

S3 Freundlich Model

The Freundlich isotherm model has the following form:

$$q_e = K_F C_e^{1/n} \qquad \text{(S4)}$$

where q_e is the adsorption capacity of contaminate (mg g^{-1}), C_e is the equilibrium concentration of contaminate in solution (mg L^{-1}); K_F and n are the Freundlich constants, which represent the adsorption capacity and the adsorption strength, respectively. The magnitude of 1/n quantifies the favorability of adsorption and the degree of heterogeneity of the adsorbent surface.

S4 Supplementary Tables and Figures

Table S1 Abbreviations that appear in this manuscript and their meanings

Abbreviation	Definition	Description
GH-1.5	GH that prepared at pH=1.5	The GO dispersion is pH=1.5, other procedure is presented in "2.1 Preparation of GH"
GH-3.5	GH that prepared at pH=3.5	The GO dispersion is pH=3.5, other procedure is presented in "2.1 Preparation of GH"
GH-5.5	GH that prepared at pH=5.5	The GO dispersion is pH=5.5, other procedure is presented in "2.1 Preparation of GH"
GH-8.5	GH that prepared at pH=8.5	The GO dispersion is pH=8.5, other procedure is presented in "2.1 Preparation of GH"
GH-12	GH that prepared at pH=12	The GO dispersion is pH=12, other procedure is presented in "2.1 Preparation of GH"
GA-1.5	GA that prepared at pH=1.5	The freeze-drying product of GH-1.5
GA-3.5	GA that prepared at pH=3.5	The freeze-drying product of GH-3.5
GA-5.5	GA that prepared at pH=5.5	The freeze-drying product of GH-5.5
GA-8.5	GA that prepared at pH=8.5	The freeze-drying product of GH-8.5
GA-12	GA that prepared at pH=12	The freeze-drying product of GH-12
V_{micro}	Pore volume of micropores	Calculated according to the HK model
V_{meso}	Pore volume of mesopores	Calculated according to the BJH and HK model
V_{macro}	Pore Volume volume of macropores	Calculated according to the BJH and HK model
SSA_{micro}	SSA of micropores	Calculated according to the SSA and BJH model
SSA_{meso}	SSA of mesopores	Calculated according to the SSA and BJH model
SSA_{macro}	SSA of macropores	Calculated according to the SSA and BJH model
M_{GH}	Mass of a GH sample	The mean value of 10 GH samples
M_{GA}	Mass of a GA sample	The mean value of 10 GA samples
M_c	Mass of confined water in a GH sample	$(M_{GH}- M_{GA}) * (I_3+I_4)$
M_b	Mass of bulk water in a GH sample	$(M_{GH}- M_{GA}) * (I_1+I_2)$
SSA_M	Total SSA normalized per GH sample	$SSA * M_{GA}$
SSA_{Mmicro}	SSA of micropores normalized per GH sample	$SSA_{micro} * M_{GA}$
SSA_{Mmeso}	SSA of mesopores normalized per GH sample	$SSA_{meso} * M_{GA}$
SSA_{Mmacro}	SSA of macropores normalized per GH sample	$SSA_{macro} * M_{GA}$
Q/OFG	Adsorption capacity normalized by content of oxygen-containing functional groups per GH sample	$Qe* M_{GA}$ / (percentage content of hydroxy and carboxyl groups)

Table S2 BET surface area and related data for GA samples

Samples	SSA	SSA_{micro}	SSA_{meso}	SSA_{macro}	Mean D	V_{micro}	V_{meso}	V_{macro}	V
GA-1.5	197.15	38.580	149.89	8.68	10.035	0.078	0.240	0.229	0.495
GA-3.5	131.55	74.05	53.20	4.31	7.837	0.055	0.111	0.111	0.258
GA-5.5	245.55	91.370	152.22	1.96	4.371	0.099	0.174	0.047	0.268
GA-8.5	311.96	127.26	183.03	1.68	4.076	0.129	0.215	0.036	0.318
GA-12	315.66	142.74	171.97	0.96	3.520	0.131	0.186	0.019	0.278

Table S3 Langmuir and Freundlich model parameters for CIP adsorption isotherms on the GH samples

Adsorbent	Langmuir			Freundlich		
	KL (L mg^{-1})	Qm (mg g^{-1})	R_2	KF	n	R_2
GH-1.5	0.0062	243.04	0.981	6.56	1.76	0.944
GH-3.5	0.0036	396.16	0.991	4.46	1.487	0.975
GH-5.5	0.0046	367.19	0.995	6.54	1.62	0.986
GH-8.5	0.0042	414.37	0.996	6.20	1.56	0.979
GH-12	0.0043	442.91	0.996	6.81	1.57	0.977

Table S4 Normalized mass, oxygen content, and SSA data

Samples	M_c	$M_{O\%}$	M_b	SSA_M	SSA_{Mmicro}	SSA_{Mmeso}	SSA_{Mmacro}
GH-1.5	0.287	0.001	0.294	1.468	0.287	1.116	0.065
GH-3.5	0.306	0.001	0.348	1.050	0.591	0.425	0.034
GH-5.5	0.315	0.001	0.497	1.883	0.701	1.167	0.015
GH-8.5	0.328	0.002	0.412	2.244	0.915	1.316	0.012
GH-12	0.409	0.003	0.325	2.589	1.171	1.410	0.008

Table S5 The regression statistics of the relationship between Q/OFG and mass of confined water and bulk water

Parameter	Value
Multiple R	0.992
R Square	0.985
Adjusted R Square	0.970
Standard Error	0.372
Observed Value	5

Table S6 Variance analysis of the relationship between Q/OFG and mass of confined water and bulk water

	df	SS	MS	F	Significance F
Regression analysis	2	17.883	8.941	64.780	0.015
Residual error	2	0.276	0.138		
Total	4	18.159			

Table S7 The regression parameters of the relationship between Q/OFG and mass of confined water and bulk water

	Coefficients	Standard error	t Stat	P-value
Intercept	-9.455	1.660	-5.695	0.029
M_b	0.002	0.002	0.945	0.444
Mc	0.045	0.004	11.376	0.008

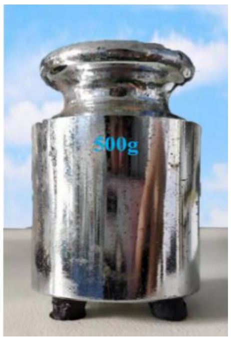

Fig. S1 500g weight supported by 3 graphene hydrogels

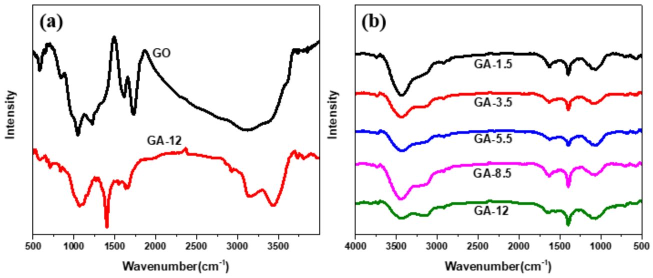

Fig. S2 FT-IR spectra of **a** GO and GA-12 and **b** GA

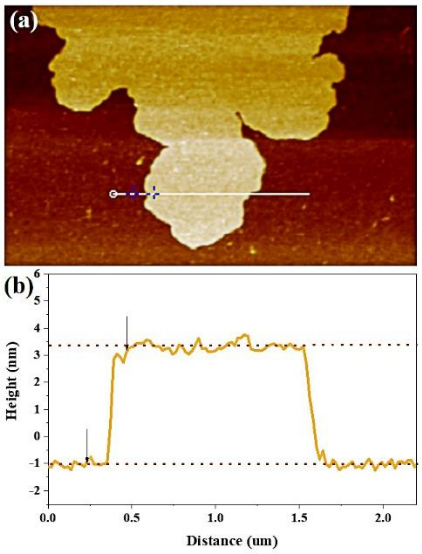

Fig. S3 a AFM image of GH-12 and **b** corresponding height profile

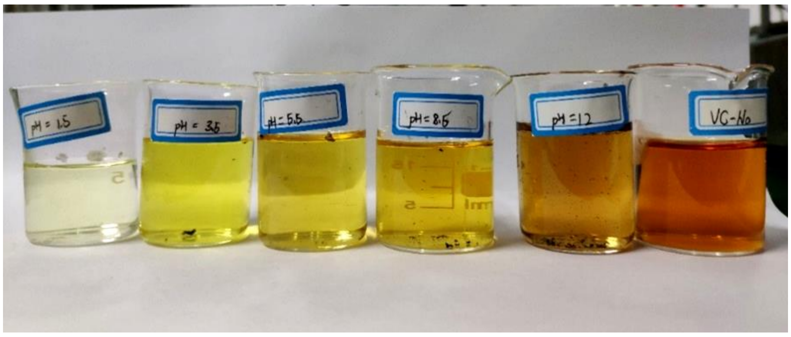

Fig. S4 Residual solutions after self-assembly process at different dispersion pH values

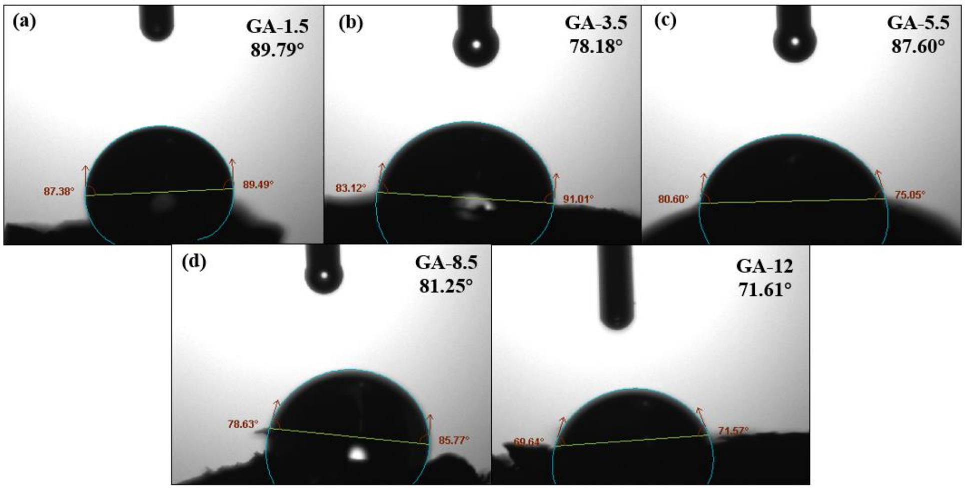

Fig. S5 a-e Contact angle of GA samples

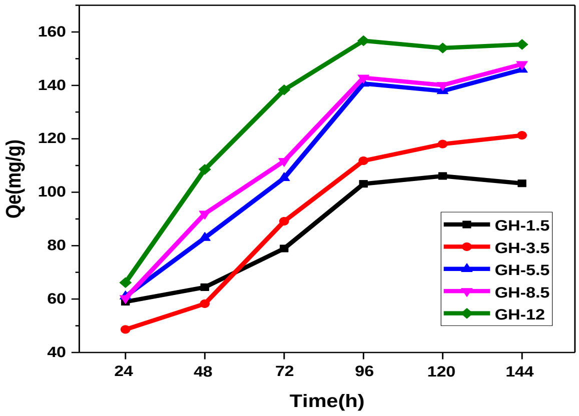

Fig. S6 Adsorption capacity of GH samples for CIP under different contact times

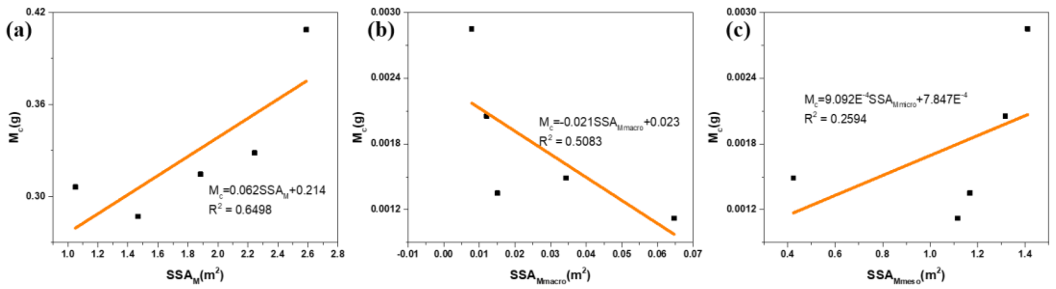

Fig. S7 The linear fitting results for **a** SSA_M (SSA of one GH sample), **b** SSAMmacro (SSA of the macropores in one GH sample), **c** SSAMmeso (SSA of the mesopores in one GH sample)

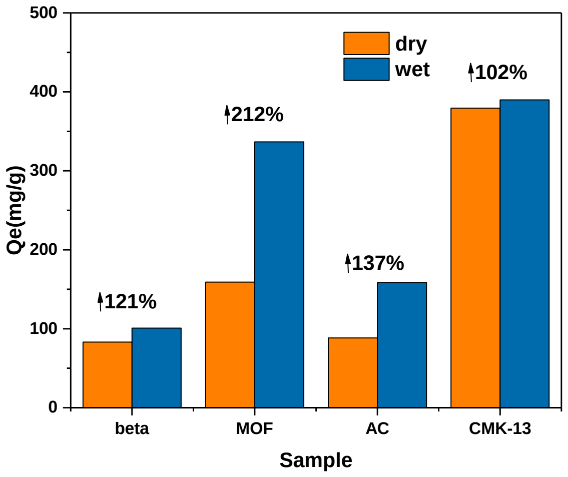

Fig. S8 The adsorption capacity of four porous adsorbents with (wet) and without (dry) confined water. The four adsorbents are Beta (SiO_2/Al_2O_3, pore diameter: 0.55-0.7nm), MOF (pore diameter: 0.8 nm), Activated Carbon (pore diameter: 2.0-2.2nm), and CMK-13 (mesoporous carbon, pore diameter: 3.8-4.0nm)
